# Phase Equilibrium and Microstructure Examinations of Eutectic Fe-C-Mn-B Alloys

**DOI:** 10.3390/ma15134393

**Published:** 2022-06-21

**Authors:** Mykhaylo Pashechko, Oleksandr Tisov

**Affiliations:** 1 Faculty of Mechanical Engineering, Politechnika Lubelska, Nadbystrzycka Str. 36, 20-618 Lublin, Poland; m.paszeczko@pollub.pl; 2 School of Aerospace Engineering, Xi’an Jiaotong University, West Xianning Road 28, Xi’an 710049, China

**Keywords:** additive manufacturing, wire arc manufacturing, phase equilibrium, Fe-C-Mn-B eutectic system, eutectic alloy, wear-resistant alloy

## Abstract

In this study, we analyzed the quaternary Fe-C-Mn-B system to create new eutectic cast alloys for coating deposition and additive manufacturing. Experimental samples were fabricated via the wire arc manufacturing method with argon shielding using Kemppi Pro 5200 Evolution equipment. Annealing was performed in a vacuum electric furnace at 1273 K for 350 h. For phase analyses, Jeol Superprobe 733 equipment was used. Metallographic and differential thermal analyses were used to reveal the eutectic structure of the samples. Examinations of the quaternary Fe-C-Mn-B system demonstrated that several eutectic alloys existed in the system. Four isothermal pseudo-ternary sections of the Fe-C-Mn-B system were studied: “Fe_3_B”-Fe_3_C-“Fe_3_Mn”; Fe_2_B-“Fe_2_C”-“Fe_2_Mn”; “Fe_3_B”-Fe_3_C-“Fe_1.2_Mn”; “Fe_23_B_6_”-“Fe_23_C_6_”-“Fe_23_Mn”. Broad eutectic concentrations enabled us to overcome parameter fluctuations during additive manufacturing. In each isothermal section, two dissimilar phase regions were determined: one with a ternary Fe-C-B composition and the other with a ternary Fe-C-Mn composition. Depending on the manganese content, two types of solid solutions could be formed: (Fe, Mn)_α_ or (Fe, Mn)_γ_.

## 1. Introduction

As natural micro- and nanocomposites, eutectic materials have long been the focus of engineers. These materials have a narrow solidification temperature range and enable the spontaneous formation of various structural cooling rate-controlled microstructures. The ductile matrix is reinforced by hard particles or lamellas formed by carbides or intermetallics. Many excellent tools and wear-resistant materials are eutectics or sintered materials with pseudo-eutectic structures. Their good casting properties also make these materials promising for additive manufacturing. Usually, the coarse microstructure formed during solidification promotes low material toughness. Fast cooling rates, however, may help to overcome this shortcoming.

Additive manufacturing developed from well-known welding processes used for decades to restore worn machine parts. The welding seam and heat-affected zone may reduce the mechanical properties of joining parts. Weld decay corrosion and weld embrittlement often result in joint failure. Recently, metal fusion during additive manufacturing has become so controlled that it is now possible to fabricate the most complex shapes with minor material consumption. It was reported that the additive-manufactured eutectic aluminum alloy AlSi_12_ has three to four times better tensile strength than conventional sand-casting [1,2]. The faster cooling rate results in higher tensile strength with minimal reduction in fatigue strength [2]. Nevertheless, both of these characteristics are remarkably higher than those of cast alloys. A faster cooling rate promotes the formation of very fine coral-like silicon eutectics in the near-eutectic alloy AlSi_10_Mg, thereby improving the material’s hardness, compressive strength, and dynamic yield. Nanoparticles also contribute to alloy strengthening. However, post-heat treatment breaks and coarsens this microstructure [3,4]. For precipitation-strengthening alloys, the as-cast and additively manufactured microstructures are very close. The additive manufacturing parameters also should be controlled. During wire arc manufacturing the torch symmetry and tilt angle directly affect the built geometry [5]. It may be adjusted by an external magnetic field. Decreasing the tilt angle reduces the powder-supported layer thus making bonding between them weak. Too big a tilt angle causes the sinking of the molten pool and the quality of the part becomes lower [6]. The study of developed forces in the built during the laser powder bed fusion process reveals significant uneven deformations caused by internal stresses reaching 80% of their maximum value [7]. It seems that for each particular case (built thickness and width, powder particle shape and average diameter) the torch or electrode tilt angle should be adjusted individually.

Various types of Fe-Ti eutectics have been investigated for use in additive manufacturing. Fe-Fe_2_Ti (17.6% Fe) eutectics can be obtained with ultra-fine microstructures and are promising for high-temperature applications under directed energy deposition [8]. This alloy has a hypereutectic composition, and phase-field modeling determined the possibility to obtain lamellar ultrafine eutectics. Later, this possibility was proven experimentally. A Ti-32.5Fe laser powder bed fusion-manufactured eutectic alloy [9] was studied, proving that rapid cooling can produce ultra-fine microstructures in the bulk material. LPBF allows one to obtain ultra-fine structures in the β-Ti/TiFe eutectic alloy, providing 30% crack-free hot compression. The oxygen intake was found to be about 0,45%. The structure of the alloy consists of a β-Ti/TiFe metallic matrix and a η-Ti4Fe_2_O_x_ dendritic micrometer size phase. This phase forms an internal frame with very high-temperature performance and bearing capacity [9].

Multicomponent near-eutectic alloys are also good candidates for additive manufacturing. Their chemical composition allows any of the properties to be altered while keeping the others constant. The near-eutectic V–9Si–5B pre-alloyed powders were fused via the directed energy deposition method [10]. Despite the alloy suffering from extra porosity, the fine eutectic structure obtained was of micrometer size. Other fine eutectic structures were obtained via laser engineering net shaping and powder-bed arc additive manufacturing of an Al_x_CoCrFeNi_2.1_ high-entropy alloy [11,12]. Increasing the aluminum content from 0.6 to 1.1% may change the eutectic type from dendritic to lamellar. The use of elemental powder also allows one to obtain homogeneous alloys with precisely controlled composition. Surprisingly, remelting of the as-manufactured alloy was found to dramatically reduce tensile strength and ductility. Repetitive reheating of the material during deposition localizes thermally-induced plasticity in the vicinity of grain boundaries. Thus, cyclic reheating of the deposited material may significantly improve the ductility of eutectic materials. This process may also help strengthen the adhesion between layers and relieve heat-induced internal stresses.

Stainless steel type 316 is one of the most widely used steels in additive manufacturing. This steel is used as a matrix for many types of materials, as well as for hybrid composites [13]. Chromium–nickel stainless steels are applied via the layer deposition method to repair cracks on metal constructions [14]. Both structural and tool steels can be used for additive manufacturing if they are stable under the selected deposition method conditions [15]. Most types of steel, especially stainless steels, do not require special shielding, as air-cooling alone produces the desired microstructure. Metal-based additive manufacturing involves the melting of powders or wires composed of the target material [16]. Powder-cored wires are also often used [17]. The compositional bond layer [18] allows one to bind steel with almost any material, including other types of steel, titanium, and nickel alloys. Thus, steel can be used in the manufacture of metal-based laminated composites.

Modern manufacturing methods can not completely avoid parameter fluctuations. For eutectic materials, the variations in eutectic concentrations are of vital importance. From this perspective, phase diagrams are indispensable for additive manufacturing materials and predetermined useful regions to control the produced material’s microstructure [13,18]. The construction of phase diagrams also helps determine the compatibility of coating and substrate materials.

Steels are often used to produce metal matrix composites reinforced by SiC, NbC, and Al_2_O_3_ microparticles. Multiple carbides can be formed in Fe-Ti and Fe-Mn systems, and iron-based amorphous materials can be obtained using fast-cooling manufacturing methods [19]. The reconditioning of worn surfaces is another important application of additive manufacturing processes. In a previous study, Co-Ni secondary hardening steels were used to recover aircraft parts via the laser deposition method [20]. However, care must be taken in the condition of the substrate. For example, reheating can cause changes in the microstructure (bainite to martensite in [21]). It is vital to consider the corrosion resistance of additively manufactured (AMed) materials, as AMed stainless steels are often superior to conventionally manufactured steels [22]. The mechanical properties of AMed stainless steels are also better than those of cast steels due to rapid cooling (10^5^–10^7^ K/s), resulting in a finer microstructure. The sizes of the inclusions and alloying element-depletion zones are also smaller. Attention should be given to avoid the formation of concentration and structural corrosion cells (e.g., on the boundaries of the molten pool).

Eutectoid iron-based alloys are extensively used in structural engineering, whereas eutectic alloys are mostly applied in the form of hardfacing coatings. However, for many applications (massive cutting tools, inserts for mills, etc.) working at high specific loads, a thick coating is not practicable. In such cases, it is better to use a bulk material. The machining of eutectic carbide-strengthened materials is troublesome due to the high hardness of such materials. Additive manufacturing produces net shape products for which only post-grinding is required. In contrast to conventional casting, this process results in a much finer microstructure and better service characteristics. Hence, it is vital to find eutectic concentrations of studied systems to produce tool alloys suitable for additive manufacturing. To produce test samples, it is better to preheat the substrate to avoid cracks [22]. A new layer deposition can reheat the sublayer, thereby providing tempering conditions. As the built material grows, this heat treatment effect becomes dissimilar in different layers; the top layer is fast-cooled, whereas the sublayers are tempered several times. The wear resistance of AMed H13 tool steels in [23] was found to be only one third that of conventionally manufactured counterparts.

The problem one encounters with additive manufacturing is that any particular material can require adjustments to the equipment [19], feedstock size and shape, feed rate, and energy input [18].

The fabrication of alloys in the form of solid solutions, chemical compounds, or eutectics requires the precise balancing of alloy components. Most metallic materials are multicomponent. Binary equilibrium diagrams are already available for a significant number of systems and have been well-described and studied [24,25,26,27]. Ternary phase diagrams are much more complicated, and quaternary systems (particularly Fe, C, Mn, and B) are much less common in the literature. There are few studies on this topic. Multiple carbides and borides present together in steel should greatly improve the cutting performance and wear resistance of the metal. To evaluate this hypothesis, we analyzed two equilibrium ternary systems, Fe-C-Mn and Fe-C-B [28,29,30]. From this review, we can figure out the eutectic regions (where full or partial eutectic exists) of elements of Fe-C-Mn and Fe-C-B systems, which will further be used to construct isothermal sections of pseudo-ternary systems and reduce the number of possible compositions (Table 1).

To produce material with the required wear resistance, the Fe_3_C, Cr_2_B, Fe_2_B, and FeB phases are very promising, as these phases are very hard, resistant to abrasion and corrosion, and thermally stable. Furthermore, the addition of manganese improves the toughness of cementite and enhances the eutectic alloy’s properties. Manganese is soluble in iron in a solid state and extends the temperature–concentration region of (Fe, Mn)_3_C mixed carbides. At the same time, manganese refines carbides and promotes their uniform distribution in the matrix material [31]. Boron partially substitutes for carbon in cementite and can form borides with iron. Manganese carbides can also be formed. The Fe-C-B and Fe-C-Mn systems are both very effective from the perspectives of mechanical properties, wear resistance, cost savings, and availability [32]. Indeed, these simple alloys have remarkable capabilities, and, as we determined, combining them into a quaternary Fe-C-Mn-B system will yield many beneficial properties.

The ternary section of this system [27] indicates that the eutectic alloy region is affected only by carbon content within a broad content range (0.2–0.8 wt%). At the same time, Mn has no significant effect, as it is infinitely soluble in iron in a solid state.

Taking into consideration the specificity and technological details of producing eutectic alloys and surface layers, as well as the obtained properties—in particular, the reduction in the tendency to crack—we chose the same carbon and boron contents used in the Fe-Mn-C eutectic alloy. The fundamental constituents of Fe-C-Mn-B, Fe, and Mn have good solubility in Ni and Cr. These elements offer strong beneficial effects on hardness, corrosion resistance, and ductility and could be used to enhance the considered eutectic system’s properties. In combinations with even more alloying elements, such as alloy steels, one can obtain versatile materials for casting, additive manufacturing, and coating deposition.

## 2. Materials and Methods

**Figure 1:**
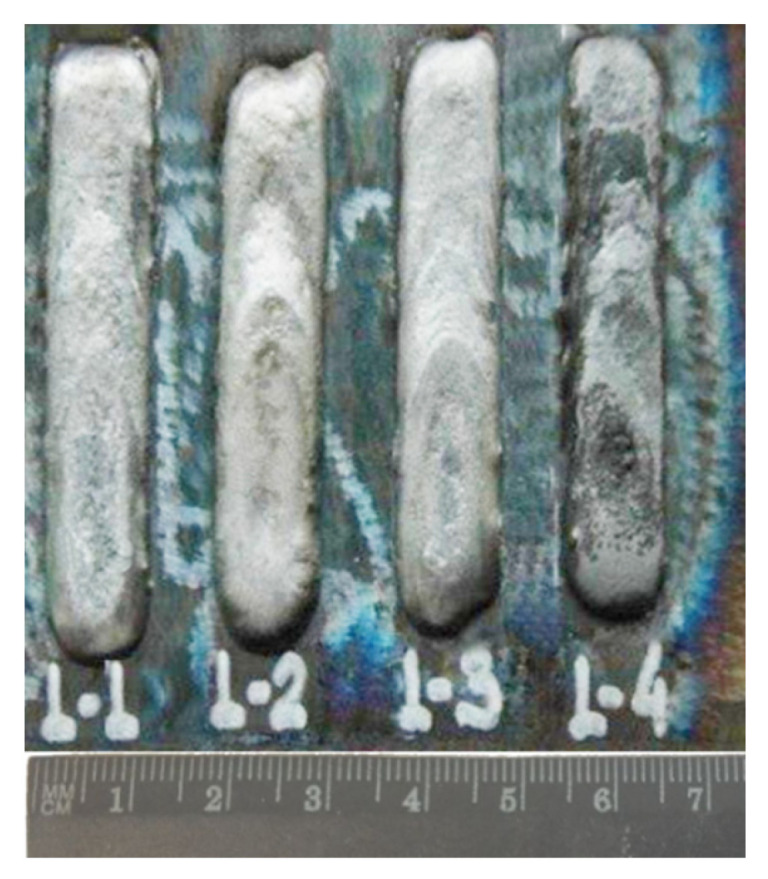
The studied alloys (##1–4, Table 1) on the base plate in as-manufactured condition.

The Fe-C-Mn-B alloys were first fabricated as powder feedstock. To fabricate the test samples of eutectic alloys to study the Fe-C-Mn-B equilibrium system, we used high purity components: Fe (99.98%), Mn (99.6%), amorphous B (99.4%), and synthetic graphite (99.95%) powders. The component concentration in each section changed with a 10 or 20 molar part pitch. Iron concentrations were in the range of 0.67–0.79 at% [28,29,30]. To prevent oxidation, minor additions of silicon were used. Silicon also enhances carbon diffusion in iron. Silicon, manganese, and boron can also effectively deoxidize steel, which is good for surface deposition technologies. Liquid metal was argon-atomized. Powders were then placed in a low-carbon (analog of AISI 1020 steel) filler wire to consider the metal’s effects on the final alloy composition. Wire arc manufacturing was done using Kemppi Pro 5200 Evolution equipment (Kemppi, Lahti, Finland) in semi-automated mode. Parameters of the manufacturing process: current = 270 A, voltage 30 V, the distance from the electrode tip to the base plate was 6 mm at the travel speed of 10 cm/min, the diameter of powder-cored wire—2.6 mm, the thickness of the weld layer 4–5 mm. To protect the alloy from oxidation, the work area was shielded by Argon gas flow (0.8 m^3^/s). The base plate (AISI 1045 steel) was preheated to 200 °C to reduce possible thermal-induced stresses. Then, samples were cut from the plate and annealed at 1273 K for 350 h. For heat treatment, the samples were enclosed in pressurized quartz containers. Figure 1 shows alloys 1–4 (Table 2), which were welded in as-manufactured condition.

Before the tests, samples were annealed (1273 K, 350 h). In order to study solid state transformations (thermal effects), we used differential scanning calorimetry (Netzsch DSC 404 F1 Pegasus) and thermal analyses (VDTA-8M; argon gas medium; maximum temperature, 1873 K; cooling rate, 80 K/min). The sample sections were polished and etched using a 3 wt.% HNO_3_ solution in ethyl alcohol. For microstructure examinations, we used Zeiss Neophot-32 (Zeiss, Jena, Germany) and MIM-8 (Lomo, St. Petersburg, Russia) light microscopes. The concentrations of chemical elements were detected using Jeol Superprobe 733 equipment (Jeol, Tokyo, Japan). A DRON-3.0 X-ray diffractometer (Burevestnik, St. Petersburg, Russia) was utilized to study the phase composition of the samples (Bragg–Brentano 2θ configuration, monochromatic Cu Kα radiation). All tests were carried out under standard atmospheric conditions.

## 3. Results and Discussion

**Figure 2:**
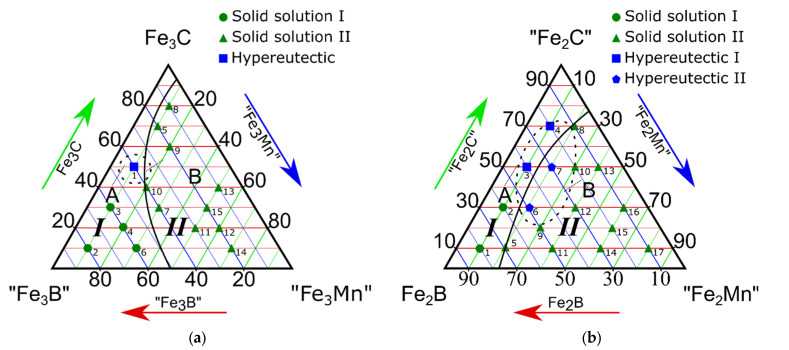

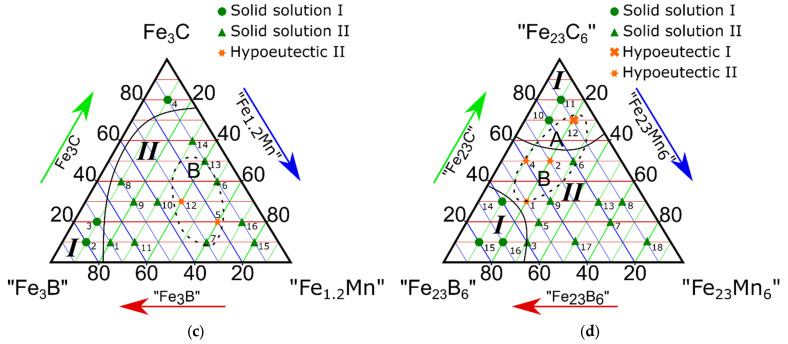
Phase regions (I, II, A, B) in isothermal sections of pseudo-ternary systems: intersections of (**a**) “Fe_3_B”-Fe_3_C-“Fe_3_Mn”, (**b**) Fe_2_B-“Fe_2_C”-“Fe_2_Mn”, (**c**) “Fe_3_B”-Fe_3_C-“Fe_1.2_Mn”, and (**d**) “Fe_23_B_6_”-“Fe_23_C_6_”-“Fe_23_Mn”.

Based on obtained results, four isothermal sections of pseudo-ternary systems were constructed: “Fe_3_B”-Fe_3_C-“Fe_3_Mn”, Fe_2_B-“Fe_2_C”-“Fe_2_Mn”, “Fe_3_B”-Fe_3_C-“Fe_1.2_Mn”, and “Fe_23_B_6_”-“Fe_23_C_6_”-“Fe_23_Mn”. The chemical compounds in quotes do not exist. These compounds were selected to build the sections from the perspective of molar ratios. The ratios of the elements were selected based on preliminary studies [26,27,30,31,32,33,34,35,36,37,38,39,40]. We combined the elements in virtual chemical compounds to simplify the graphical representations of the quaternary to ternary phase diagrams. Each section has two phase regions, I and II, and eutectic regions A (in phase region I) and B (in phase region II) (Figure 2a–d).

**Figure 3:**
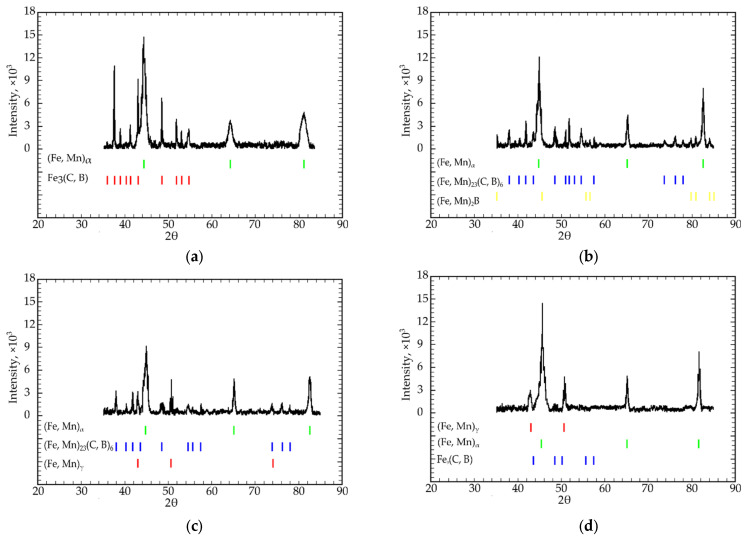

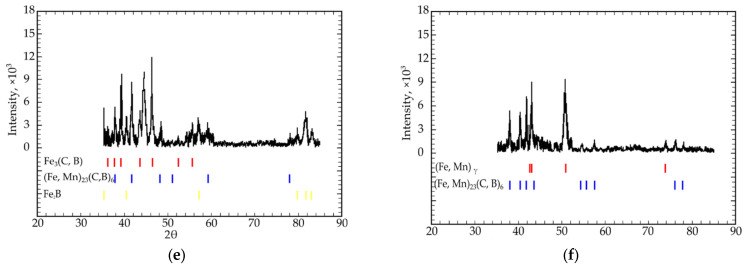
Common XRD patterns of the studied alloys (Table 2, Table 3, Table 4 and Table 5). (**a**) alloy consists of alloyed by manganese ferrite (Fe, Mn)_α_ and particles of cementite alloyed by boron (#3 Table 2), (**b**) alloy contains (Fe, Mn)_α_ solid solution and particles of (Fe, Mn)_2_B boride, and (Fe, Mn)_23_(C, B)_6_ borocarbide (#8 Table 3), (**c**) alloy #10 Table 5 contains two solid solutions: alloyed ferrite and alloyed austenite with particles of borocarbide (Fe, Mn)_23_(C, B)_6_, (**d**) alloy 13, Table 5 contains alloyed ferrite and austenite with the particles of boron-alloyed cementite Fe_3_(C, B), (**e**) diffraction pattern of alloy 6 from Table 3 contains alloyed cementite Fe_3_(C, B), borocarbide (Fe, Mn)_23_(C, B)_6_ and alloyed iron boride (Fe, Mn)_2_B, (**f**) alloy 15, Table 4 consists of borocarbide (Fe, Mn)_23_(C, B)_6_ particles in (Fe, Mn)_γ_ solid solution.

Results of X-ray diffraction examinations were used to determine phase analyses of the samples. Based on these results, Table 1, Table 2, Table 3 and Table 4 were composed. Basically, the X-ray diffraction patterns most common for this study are presented in Figure 3a–f. The analyses of XRD patterns allow to state on the existence of two basic solid solutions in the system: Fe_α_ and Fe_γ_ alloyed by Mn, designated as (Fe, Mn)_α_ and (Fe, Mn)_γ_, respectively. Commonly, only one of them is present, but in some alloys (Table 2, ##11, 12, Table 5, ##10, 13, and other compositions) they may exist simultaneously. In addition, traces of unalloyed (or with an undetectable amount of alloying elements) Fe_α_ were detected (Table 3, ##1–5, Table 5, #11). This is the evidence of the partitioning of alloying elements (or segregation) which was studied by authors on other alloy systems [34,36].

In the Fe-C system, if alloying elements are added, the cementite structure may be modified. Particularly in the considered system, iron atoms may be replaced by manganese, and carbon atoms by boron [36]. Thus, this constituent is called alloyed cementite and we designate it as Fe_3_(C, B) (Figure 3a,d,e). In other phase regions (alloys ##8, 10–12 in Table 2, 9–17 in Table 3 and others) more complex borocarbide is formed: (Fe, Mn)_23_(C, B)_6_.

In alloys with a higher concentration of boron and lower carbon content, iron borides are formed: Fe_2_B (Figure 3e, alloys 6 and 9, Table 3). In the majority of alloy compositions, boron does not form a separate chemical compound, but a part of alloyed cementite and borocarbide. In alloy compositions 8 and 9 (Table 2) alloyed iron boride was detected (Figure 3b) as a minor constituent

**Figure 4:**
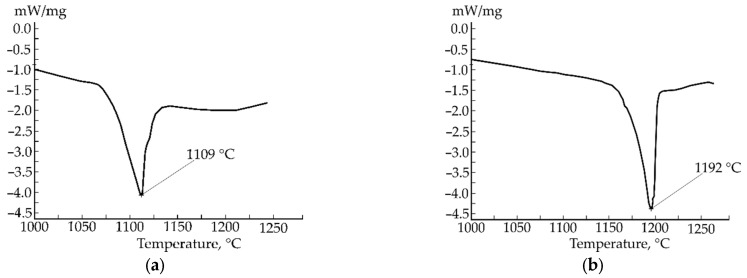
DSC curves of eutectic alloys of Fe-C-Mn (**a**) and Fe-C-B (**b**) subsystems.

The DSC curves were used to check the assumption about the eutectic concentration of the alloy. This was done based on the property of eutectic alloy to melt at a definite temperature. Typically, this temperature is the lowest in the system, but in our case of the quaternary system, we assumed the formation of Fe-C-Mn and Fe-C-B type eutectics. The DSC curves indicating the eutectic temperature of these separate ternary subsystems within a quaternary Fe-C-Mn-B system are presented in Figure 4. According to our results, the melting point of Fe-C-Mn type eutectic is in the region of 1192 °C, which is in good agreement with [37], and the melting point of Fe-C-B type eutectic was estimated at 1109 °C. Thus, using DSC, the existence of two separate eutectic regions was proved. Nevertheless, we should take in mind that these temperatures may be affected by the presence of admixture atoms (B in the case of Fe-C-Mn type eutectic and Mn in Fe-C-B type eutectic).

### 3.1. Study on the Isothermal Section of the “Fe_3_B”-Fe_3_C-“Fe_3_Mn” Pseudo-Ternary System

Studying the “Fe_3_B”-Fe_3_C-“Fe_3_Mn” isothermal pseudo-ternary section evidenced two dissimilar phase regions (Figure 2a; Table 2). Phase region I consisted of (Fe, Mn)_α_ and (Fe, Mn)_γ_ solid solutions and Fe_3_(C, B) iron borocarbide. Phase region II contained (Fe, Mn)_α_ and (Fe, Mn)_γ_ solid solutions, (Fe, Mn)_2_B boride, and (Fe, Mn)_23_(C, B)_6_ multicomponent borocarbide.

Hypereutectic alloys had the following composition: iron, 67.8–75.0; manganese, 1.2–4.5; boron, 8.5–14.3; carbon, 11.0–13.9 C (in at%) [9,13].

**Figure 5:**
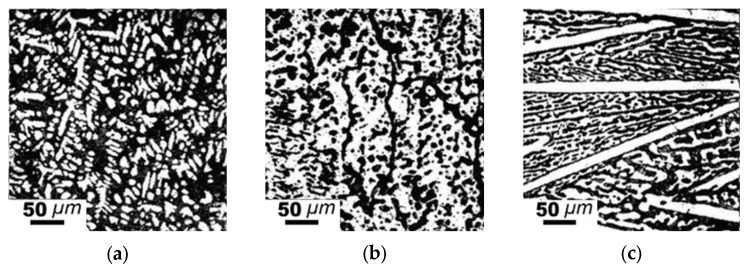
Microstructure of hypereutectic alloys #3 (**a**,**b**) and #8 (**c**); (**a**) as-cast; (**b**,**c**) annealing at 1273 K, 350 h; alloy designation is the same as that in Table 2. Here and on other following microstructures, carbide particles—light field, solid solution—dark field.

The 19 samples of this pseudo-ternary equilibrium system were then examined. Three constituents were determined: cementite, (Fe, Mn)_23_(C, B)_6_, and (Fe, Mn)_3_(C, B) phases with the structure of Fe_23_C_6_ and Fe_3_C. Samples ##6 and 10 presented phases with a structure of cementite and the following lattice parameters: a = 0.5091, b = 0.6650, and c = 0.4559 nm. The lattice parameters of this cementite-like structure corresponded to Fe_3_C. Samples ##1–3, 5, and 7 contained the Fe_3_C phase with increased lattice parameters. This phase was identified as borocarbide Fe_3_(C, B). Many of the samples consist of solid solution, (Fe, Mn)_23_(C, B)_6_ phase and/or Fe_2_B, (Fe, Mn)_2_B borides. Samples ##1–14 (Table 2) contained a solid solution of (Fe, Mn)_α_. It also should be mentioned that in phase region I (Figure 2a), a hypereutectic structure was achieved for alloy #3 with a composition of Fe_75_C_12.5_Mn_2.5_B_10_ (at.%). It consists of dendritic (Fe, Mn)_23_(C, B)_6_ carbides (light field) and (Fe, Mn)_α_ + Fe_3_(C, B) eutectic (dark field) between them (Figure 5a). These dendrites are oriented along the basic crystallographic directions [36]. High-temperature annealing at 1273 K during 350 h led to carbides coarsening (Figure 5b). In carbon-rich alloys, the (Fe, Mn)_23_(C, B)_6_ compound occurred in the form of lamellas that were 250–450 μm in length and 10–30 μm in width. Carbide lamellas as long as the studied sample were also identified (Figure 5c).

Eutectic carbide particles grew from these lamellas and ran parallel to each other. The microstructure here is dendritic. All other alloys in phase regions I and II were of the solid solution type and consisted of (Fe, Mn)_α_ and (Fe, Mn)_γ_ solid solutions containing (Fe, Mn)_2_B boride, Fe_3_(C, B), or (Fe, Mn)_23_(C, B)_6_ borocarbide particles. The Fe-C-B type eutectic was located in region A, whereas the Fe-C-Mn type eutectic was in region B (Figure 2a).

### 3.2. Study on the Isothermal Section of the Fe_2_B-“Fe_2_C”-“Fe_2_Mn” Pseudo-Ternary System

**Figure 6:**
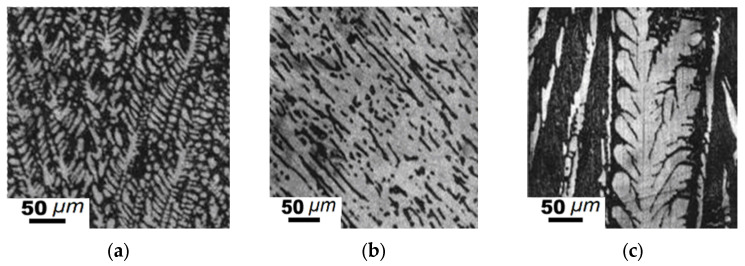
Microstructure of alloys of the Fe_2_B-“Fe_2_C”-“Fe_2_Mn” pseudo-ternary section (designations are the same as those in Table 4): (**a**,**b**) sample #3, (**c**) sample #10; (**a**,**c**) as cast; (**b**) annealed 350 h at 1273 K.

The sample composition and phases detected in the pseudo-ternary Fe_2_B-“Fe_2_C”-“Fe_2_Mn” system (Figure 2b) are listed in Table 3. The alloys contained the (Fe, Mn)_γ_ solid solution and Fe_3_(C, B), (Fe, Mn)_23_(C, B)_6_, Fe_2_B chemical compounds. The iron content for these samples was 66.6 at.%. Here, we separated the two phase regions (Figure 2b; Table 3). Region I contained Fe_3_(C, B) particles in the Fe_α_ solid solution. Hypereutectic region II contained (Fe, Mn)_γ_ and (Fe, Mn)_23_(C, B)_6_. The Fe-C-B type eutectic alloy was located in field A, whereas the Fe-C-Mn type eutectic was in field B (Figure 2b). Hypereutectic alloys contained 66.6 at.% of iron and 3.3–6.7 at.% of manganese, as well as 10.0–23.3 at.% of carbon and 6.7–16.6 at.% of boron. Hypereutectic samples ##3–4 contained primary Fe_3_(C, B) crystals and dendrites of (Fe, Mn)_23_(C, B)_6_ iron-manganese borocarbide (Figure 6a,b).

In boron-rich alloys, the formation of primary austenite crystals was detected. In eutectic (Fe, Mn)_γ_-(Fe, Mn)_23_(C, B)_6_ alloys, we observed micro-size fields of the (Fe, Mn)γ solid solution and dendrites of austenite. In alloys with an elevated manganese concentration, primary (Fe, Mn)_23_(C, B)_6_ dendrites were formed (Figure 6c). The composition of the eutectic alloys was as follows: 66.6 at.% Fe, 7.7–25.0 at.% C, 1.7–11.0 at.% Mn, and 4.0–8.4 at.% B [4,12].

### 3.3. Study on the Isothermal Section of the “Fe_3_B”-Fe_3_C-“Fe_1.2_Mn” Pseudo-Ternary System

In the studied pseudo-ternary section, most of the “Fe_3_B”-Fe_3_C-“Fe_1.2_Mn” samples (Figure 2c) consisted of the (Fe, Mn)_23_(C, B)_6_ phase in equilibrium with other iron-containing phases, specifically Fe_3_(C, B) and (Fe, Mn)_γ_. Phase analyses revealed the (Fe, Mn)_γ_ + Fe_3_(C, B) + (Fe, Mn)_23_(C, B)_6_ phase region [31,32]. Samples did not contain Fe_α_. The phase composition of the samples is given in Table 4.

According to the micro X-ray and microstructural analyses performed for the “Fe_3_B”-Fe_3_C-“Fe_1.2_Mn” pseudo-ternary section, two phase regions were identified (Figure 2c; Table 4). Region I contained the Fe_3_(C, B), (Fe, Mn)_23_(C, B)_6_, and (Fe, Mn)_γ_ phases. Region II consisted of the (Fe, Mn)_γ_, (Fe, Mn)_23_(C, B)_6_, and Fe_3_(C, B) phases. Hypoeutectic alloys were located in region II (Figure 2c). The examined section area B is enriched by manganese, where the Fe-C-Mn eutectic was detected (Figure 2c).

**Figure 7:**
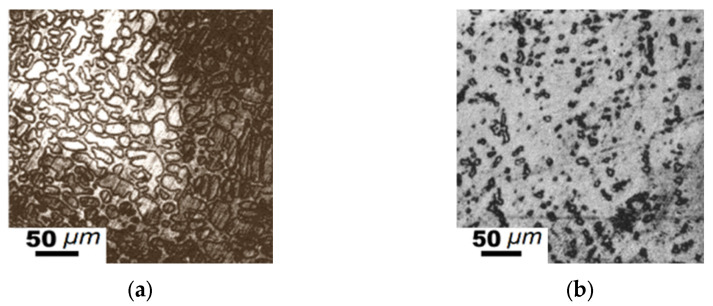
Microstructure of the studied “Fe_3_B”-Fe_3_C-“Fe_1.2_Mn” alloys (Table 5): (**a**) #5, hypoeutectic alloy, normalized; (**b**) solid solution, #6 annealed at 1273 K for 350 h.

When Mn content in the hypoeutectic alloys increased, the primary dendrites of (Fe, Mn)_γ_ formed first. The eutectic consisting of (Fe, Mn)_γ_ and (Fe, Mn)_23_(C, B)_6_ was located in the areas between these dendrites (Figure 7a). After high-temperature annealing, these microstructures of the alloys presented homogenized and coagulated (Fe, Mn)_γ_ crystals (Figure 7b). The contents of the components in these samples were as follows: Fe—65.3–71.1, Mn—8.7–21.5, C—2.8–15.4, and B—2.9–10.4 (at%).

### 3.4. Study on the Isothermal Section of the “Fe_23_B_6_”-“Fe_23_C_6_”-“Fe_23_Mn” Pseudo-Ternary System

An isothermal section of the “Fe_23_B_6_”-“Fe_23_C_6_”-“Fe_23_Mn” in the pseudo-ternary system is given in Figure 2d. Sample #3 contains the largest number of equilibrium phases, with (Fe, Mn)_γ_, Fe_3_(C, B), (Fe, Mn)_23_(C, B)_6_, and (Fe, Mn)_α_. Phase region II, alloy #10, contained the largest number of phases, with Fe_3_(C, B) + a minor amount of (Fe, Mn)_α_ + (Fe, Mn)_γ_. Samples ##1 and 10–13 (Table 5) presented a ternary phase of Fe_3_(C, B) [32]. Many alloys contained a four-component phase of (Fe, Mn)_23_(C, B)_6_. For this isothermal section, the average Fe content was 79 (at.%). The phase equilibrium of (Fe, Mn)_α_ + (Fe, Mn)_γ_ + Fe_3_(C, B) + (Fe, Mn)_23_(C, B)_6_ was also identified (#3). The (Fe, Mn)_γ_ solid solution phase was observed in Samples ##3, 6–8, 12, and 13. The equilibrium of (Fe, Mn)_α_ and (Fe, Mn)_γ_ was observed in alloys ##3, 12, and 13 (Table 5).

Micro X-ray and microstructural examinations revealed two dissimilar phase regions (Figure 2d; Table 6). Region I contained (Fe, Mn)_α_, (Fe, Mn)_γ_, Fe_3_(C, B), and Fe_α_. Region II contained (Fe, Mn)_23_(C, B)_6_ and Fe_3_(C, B) chemical compounds alongside (Fe, Mn)_α_ and (Fe, Mn)_γ_ solid solutions. The Fe-C-B type eutectic alloys were located in region A, whereas the Fe-C-Mn type eutectic structures were localized in region B (Figure 2d).

**Figure 8:**
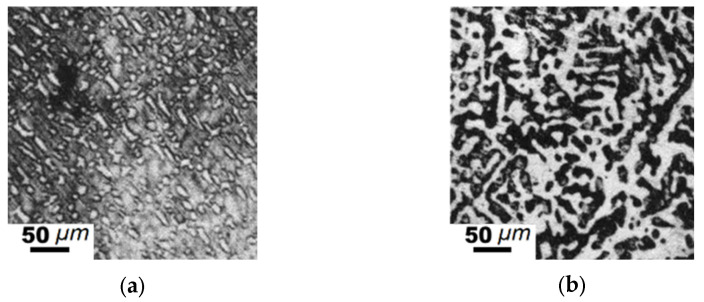
Microstructure of the hypoeutectic alloy for Sample #1 (according to Table 5): (**a**) as cast; (**b**) annealed at 1273 K for 350 h.

The element concentration in hypoeutectic alloys was determined next (at.%), with 79.3% Fe, 2.7–6.2% Mn, 5.4–15.5% of C, and 1.1–10.8% B. Austenite dendrites were formed under elevated carbon contents, whereas pearlite dendrites were formed at lower carbon concentrations (Figure 8a). Austenite dendrites were uniformly distributed in the alloy. After annealing, much coarser crystals of (Fe, Mn)_γ_ appeared (Figure 8b).

Comparing the results for the four isothermal sections, we observed that the iron–manganese borocarbide phase (Fe, Mn)_23_(C, B)_6_ was present in all pseudo-ternary systems. A separate phase was observed in alloys with an iron concentration of 79 at% (molar fraction): [40“Fe_23_B_6_”-0“Fe_23_C_6_”-10“Fe_23_Mn_6_”]-[50“Fe_3_B”-40Fe_3_C-10“Fe_1.2_Mn”] and the isothermal section “Fe_3_B”-Fe_3_C-“Fe_1.2_Mn”.

(Fe, Mn)_23_(C, B)_6_ borocarbide was detected as the primary phase in [(10-50)Fe_2_B-(10-50)“Fe_2_C”-40“Fe_2_Mn”]-[(20-30)Fe_2_B-(20-50)“Fe_2_C”-(20-60)“Fe_2_Mn”] alloys at an iron concentration of 66.6 at.%, the same as the results observed for [(30-40)“Fe_3_B”-(20-30)Fe_3_C-(40-50)“Fe_3_Mn”]-[10“Fe_3_B”-2Fe_3_C-80“Fe_3_Mn”] alloys at iron concentrations of 75 at.%. The relation here is clear: when iron content reduces, the boron and manganese content in (Fe, Mn)_23_(C, B)_6_ increases, and carbon content decreases (Figure 2a,b). The hypereutectic concentrations of the system are outlined in Figure 2 and Table 6.

The studied eutectic alloys were dispersion-strengthened natural composites consisting of relatively soft matrix phase: alloyed austenite or pearlite, strengthened with a hard and wear-resistant (Fe, Mn)_23_(C, B)_6_ phase. By changing the contents of the alloying elements, it is possible to obtain two or more phases in equilibrium [17,38,39,40,41,42,43,44,45,46,47,48,49,50]. By balancing the four components, one can obtain an alloy with the required combination of carbide content and composition. Eutectic and hypereutectic alloys have good casting properties and do not form cracks when wire arc-manufactured. This material, moreover, can be modified by other alloying elements to enhance its properties.

## 4. Conclusions

Wire arc-manufactured alloy samples were found to be free of cracks, and the thickness of the built products was nearly identical. XRD analyses also demonstrated that the chemical composition of the alloys was nominal. Microstructural studies revealed different eutectic structures depending on chemical composition. This fact could allow one to change the microstructure according to the requirements of any casting or additive manufacturing process, not only wire arc manufacturing.

The main results are listed below:

- Fe-C-B type eutectic alloys were formed with a manganese content of 1.6–7.6 wt.%. The concentration of the other components was 85.1–92.5 wt.% for iron, 2.6–7.0 wt.% for carbon, and 0.2–3.5 wt.% for boron;
- Fe-C-Mn type eutectic alloys were formed with a boron content of 0.6–2.5 wt.%. The concentrations of the other alloying elements were 73.3–92. 5 wt.% for iron, 3.1–23.8 wt.% for manganese, and 0.6–6.4 wt.% for carbon;
- All four isothermal sections consisted of two different phase regions composed of ternary Fe-C-B (region I) and Fe-C-Mn (region II) systems (Figure 2a–c). Region I consisted of a combination of the following phases: Fe_3_(C, B) + (Fe, Mn)_α_ + (Fe, Mn)_γ_, and region II consisted of (Fe, Mn)_γ_ + (Fe, Mn)_α_ + (Fe, Mn)_23_(C, B)_6_;
- The alloys of the “Fe_3_B”-Fe_3_C-“Fe_1.2_Mn” isothermal section at 66.6 at.% for iron contained no α-phase. This result was only observed when the iron content was higher than 75 at.%;
- In the eutectic region with the highest Fe concentration (79; 75 at.%), the (Fe, Mn)_γ_, (Fe, Mn)_α_, Fe_3_(C, B), and (Fe, Mn)_23_(C, B)_6_ phases were in equilibrium. When the iron concentration reduced to 66.6 at.% (the “Fe_3_B”-Fe_3_C-“Fe_1.2_Mn” isothermal section), the alloys presented no γ-α transition in the Fe-based solid solution. In this case, Fe_3_(C, B) + (Fe, Mn)_γ_ or (Fe, Mn)_23_(C, B)_6_ + (Fe, Mn)_γ_ were in equilibrium;
- The eutectic regions in the isothermal sections of the “Fe_3_B”-Fe_3_C-“Fe_3_Mn”, Fe_2_B-“Fe_2_C”-“Fe_2_Mn”, and “Fe_23_B_6_”-“Fe_23_C_6_”-“Fe_23_Mn” pseudo-ternary systems were found in both phase regions. The “Fe_3_B”-Fe_3_C-“Fe_1.2_Mn” system featured a eutectic Fe-C-Mn structure in region II only. Basically, the Fe-C-B type eutectic was formed when the boron and carbon contents were high, whereas the Fe-C-Mn type eutectic was found in the region with a high Mn concentration.
- It was experimentally proved that in the Fe-C-Mn-B quaternary system there are two separate eutectic regions—Fe-C-B and Fe-C-Mn—and they have the same concentrations, as it is in respective ternary systems. The knowledge of this is fundamental in the development of new eutectic alloys based on the quaternary Fe-C-Mn-B system.

## Figures and Tables

**Table 1:** Contents of elements in the eutectic regions of Fe-C-Mn and Fe-C-B systems [10].

Element	Fe-C-Mn wt.%		Fe-C-B wt.%	
	Min	Max	Min	Max
Fe	73.3	92.5	85.1	92.5
C	0.6	6.4	2.6	7.0
Mn	3.1	23.8	1.6	7.6
B	0.6	2.5	0.2	3.5

**Table 2:** Phase composition of Fe-C-Mn-B samples (isothermal section of “Fe_3_B”-Fe_3_C-“Fe_3_Mn” system (Figure 2a)).

	Component Contents, Molar Fractions			Phase Composition	Alloy Type	Phase Region (Figure 2a)
#	“Fe_3_B”	Fe_3_C	“Fe_3_Mn”			
1	40	50	10	(Fe, Mn)γ + (Fe, Mn)_α_ + Fe_3_(C, B)	Hypereutectic alloy	I
2	80	10	10	(Fe, Mn)_α_ + Fe_3_(C, B)	Solid solution	
3	60	30	10			
4	60	20	20			
5	20	70	10	(Fe, Mn)_α_ + Fe_3_(C, B)		
6	60	10	30			
7	40	30	30			
8	10	80	10	(Fe, Mn)_α_ + (Fe, Mn)_23_(C, B)_6_ + (Fe, Mn)_2_B	Hypereutectic	II
9	20	60	20			
10	40	40	20	(Fe, Mn)_α_ + (Fe, Mn)_γ_ + (Fe, Mn)_23_(C, B)_6_	Solid solution	
11	30	20	50			
12	20	20	60			
13	10	40	50	(Fe, Mn)_γ_ + (Fe, Mn)_α_ + (Fe, Mn)_23_(C, B)_6_		
14	20	10	70			
15	20	30	50	(Fe, Mn)_γ_ + (Fe, Mn)_23_(C, B)_6_		

**Table 3:** Phase composition of Fe-C-Mn-B samples with iron content of 66.6 at.% (isothermal section of the Fe_2_B-“Fe_2_C”-“Fe_2_Mn” system (Figure 2b)).

	Component Contents, Molar Fractions			Phase Composition	Alloy Type	Phase Region (Figure 2b)
#	Fe_2_B	“Fe_2_C”	“Fe_2_Mn”			
1	80	10	10	Fe_α_ + Fe_3_(C; B)	Solid solution	I
2	60	30	10			
3	40	50	10		Hypereutectic alloy	
4	20	70	20			
5	70	10	20		Solid solution	II
9	50	20	30	Fe_3_(C, B) + (Fe, Mn)_23_(C, B)_6_ + Fe_2_B		
6	50	30	20		Hypereutectic alloy	
7	30	50	20	(Fe, Mn)_γ_ + (Fe, Mn)_23_(C, B)_6_		
10	20	50	30	(Fe, Mn)_γ_ + (Fe, Mn)_23_(C, B)_6_	Solid solution	
11	50	10	40			
12	30	30	40			
13	10	50	40			
15	20	20	60			
14	30	10	60	(Fe, Mn)_γ_ + (Fe, Mn)_23_(C, B)_6_		
16	10	30	60			
17	10	10	60			
8	10	70	20	(Fe, Mn)_γ_ +		

**Table 4:** Phase composition of Fe-C-Mn-B samples and isothermal section of the “Fe_3_B”-Fe_3_C-“Fe_1.2_Mn” pseudo-ternary system (Figure 2c).

	Component Contents, Molar Fractions			Phase Composition	Alloy Type	Phase Region (Figure 2c)
#	“Fe_3_B”	Fe_3_C	“Fe_1.2_Mn”			
2	80	10	10	(Fe, Mn)_γ_ + Fe_3_(C, B)	Solid solution	I
3	70	20	10	Fe_3_(C, B)		
4	10	80	10			
1	70	10	20	(Fe, Mn)_23_(C, B)_6_ + Fe_3_(C, B)	Solid solution	II
5	20	20	30	(Fe, Mn)_γ_ + (Fe, Mn)_23_(C, B)_6_	Hypoeutectic	
12	30	30	40			
6	10	40	50		Solid solution	
7	30	10	60			
8	50	40	10			
9	50	30	20			
10	40	30	30			
11	60	10	30			
13	10	50	40			
14	10	60	30	(Fe, Mn)_γ_ + (Fe, Mn)_23_(C, B)_6_ +		
15	10	10	80			
16	10	20	70	(Fe, Mn)_γ_ + (Fe, Mn)_23_ (C, B)_6_		

**Table 5:** Phase composition of Fe-C-Mn-B samples and an isothermal section at an iron concentration of 79 at.%.

	Component Contents, Molar Fractions			Phase Composition	Alloy Type	Phase Region (Figure 2d)
#	“Fe_23_B_6_”	“Fe_23_C_6_”	“Fe_23_Mn_6_^”^			
1	50	30	20	(Fe, Mn)_α_ + (Fe, Mn)_23_(C, B)_6_ + Fe_3_(C, B)	Hypoeutectic	II
2	30	50	20	Fe_α_ + (Fe, Mn)_23_(C, B)_6_		
4	40	50	10	(Fe, Mn)_23_(C, B)_6_		
3	60	10	30	(Fe, Mn)_γ_ + (Fe, Mn)_α_ + Fe_3_(C, B) + (Fe, Mn)_23_(C, B)_6_	Solid solution	
5	40	20	30	(Fe, Mn)_γ_ + (Fe, Mn)_23_(C, B)_6_		
6	20	50	30			
7	20	20	60	(Fe, Mn)_γ_ + (Fe, Mn)_23_(C, B)_6_		
8	10	30	60			
13	20	30	50	(Fe, Mn)_γ_ + (Fe, Mn)_α_ + Fe_3_(C, B)		
9	40	30	30	(Fe, Mn)_γ_ + (Fe, Mn)_23_(C, B)_6_	Solid solution	I
17	40	10	50			
18	10	10	80			
10	20	70	10	(Fe, Mn)_α_ + (Fe, Mn) _γ_ + (Fe, Mn)_23_(C, B)_6_		
11	10	80	10	Fe_α_ + Fe_3_(C, B)		
12	10	70	20	(Fe, Mn)_α_ + (Fe, Mn)_γ_ + Fe_3_(C, B)	Hypoeutectic	
14	60	30	10	(Fe, Mn)_γ_ + (Fe, Mn)_23_(C, B)_6_ + Fe_3_(C, B)		
15	80	10	10			
16	70	10	20			

**Table 6:** The content of alloying elements in eutectic regions of “Fe_3_B”-Fe_3_C-“Fe_3_Mn”, Fe_2_B-“Fe_2_C”-“Fe_2_Mn”, “Fe_3_B”-Fe_3_C-“Fe_1.2_Mn”, and “Fe_23_B_6_”-“Fe_23_C_6_”-“Fe_23_Mn” isothermal sections of the Fe-C-Mn-B system (* first row: wt.%, ** second row: at.%).

Pseudo-Ternary Section		Content of Elements							
		Fe		C		Mn		B	
		min	max	min	max	min	max	min	Max
System	Fe-C-Mn-B	73.3 65.3	92.5 * 79.3 **	0.6 2.8	7.0 25.0	1.6 1.2	23.8 21.5	0.2 1.1	3.5 18.4
Eutectic	Fe-C-Mn	73.3 65.3	92.5 79.3	0.6 2.8	6.4 23.4	3.1 2.7	23.8 21.5	0.6 2.9	2.5 18.4
Eutectic	Fe-C-B	85.1 66.6	92.5 79.3	2.6 10.5	7.0 25.0	1.6 1.2	7.6 6.2	0.2 1.1	3.5 17.7
Section	Fe_2_B-“Fe_2_C”-“Fe_2_Mn”	81.2 66.6	89.2	2.0 7.7	7.0 25.0	2.2 1.7	13.1 11.0	0.9 4.0	4.5 8.4
Eutectic	Fe-C-Mn	81.2 66.6	86.3	2.0 7.7	6.4 23	6.0 4.7	13.1 11.0	0.9 4.0	4.6 18.4
Eutectic	Fe-C-B	85.1 66.6	89.2	3.0 11.0	7.0 25.0	2.2 1.7	7.6 6.0	0.9 4.0	4.4 17.7
Intersection	“Fe_3_B”-Fe_3_C-“Fe_3_Mn”	87.1– 67.8–	92.5 75.0	2.8 11.0	3.7 13.9	1.6 1.2	5.7 4.5	1.9 8.5	3.5 14.3
Eutectic	Fe-C-Mn	90.1 75.0	91.2	2.8 11.0	3.5 13.7	3.3 2.8	4.8 4.0	1.9 8.5	2.4 10.0
Eutectic	Fe-C-B	87.1 67.8	92.5 75.0	2.8 11.0	3.7 13.9	1.6 1.2	5.7 4.5	2.0 8.5	3.5 14.3
Intersection	“Fe_3_B”-Fe_3_C-“Fe_1,2_Mn”	73.3 –	84.9 –	0.6 –	0.4 –	10.2 –	23.8 –	0.6 –	2.3 –
Eutectic	Fe-C-Mn	65.3 –	71.1 –	2.8	15.4 –	8.7 –	21.5 –	2.9 –	10.4 –
Intersection	“Fe_23_B_6_”-“Fe_23_C_6_”-“Fe_23_Mn_6_”	89.7 79.3	92.5	1.4 5.4	3.9 15.5	3.1 2.7	6.9 6.2	0.2 1.1	2.5 10.8
Eutectic	Fe-C-Mn	89.7 79.3	92.5	1.4 5.4	2.9 10.9	3.1 2.7	6.9 6.2	0.7 3.6	2.5 10.8
Eutectic	Fe-C-B	89.7 79.3	92.5	2.8 10.7	3.9 15.5	3.1 2.7	6.7 6.1	0.2 1.1	1.5 6.8

## Data Availability

The data that support the findings of this study are available from the corresponding author upon reasonable request.
